# Cationic nano-copolymers mediated IKKβ targeting siRNA to modulate wound healing in a monkey model of glaucoma filtration surgery

**Published:** 2010-11-26

**Authors:** Hehua Ye, Yiyong Qian, Mingkai Lin, Yongheng Duan, Xuerong Sun, Yehong Zhuo, Jian Ge

**Affiliations:** 1 State Key Laboratory of Ophthalmology, Zhongshan Ophthalmic Center, Sun Yat-sen University, Guangzhou, P.R. China; 2 Department of Ophthalmology, First Affiliated Hospital of Soochow University, Suzhou, P.R. China; 3 Physical Education Science School, Zhanjiang Normal College, Zhanjiang, P.R. China

## Abstract

**Purpose:**

To investigate the efficacy and safety of cationic nano-copolymers CS-*g*-(PEI-*b*-mPEG) mediated IκB kinase beta (IKKβ) targeting siRNA in modulating wound healing in a monkey model of glaucoma filtration surgery.

**Methods:**

The IKKβ targeting siRNAs were chemically synthesized and screened in cultured monkey Tenon’s fibroblasts in vitro. Fourteen monkeys underwent trabeculectomy and were randomly allocated to one of three treatment regimens: subconjunctival injection of either CS-*g*-(PEI-*b*-mPEG)/IKKβ-siRNA (six eyes, 50nM, at the time of surgery and 7 days post surgery) or phosphate buffered saline (four eyes), or treated with mitomycin C (MMC; four eyes, 0.2 mg/ml). Bleb survival and characteristics, and intraocular pressure, were evaluated over a 60-day period. Histology of the surgical eyes was performed to evaluate ocular scarring and fibrosis in each group.

**Results:**

Subconjunctival injection of CS-*g*-(PEI-*b*-mPEG)/IKKβ-siRNA was well tolerated in this model. Both siRNA and MMC significantly prolonged bleb survival compared with the PBS group (the medians for survival days were 45.5, 60, and 29.5 in the siRNA, MMC, and PBS groups, respectively, p<0.01). Higher blebs were observed in the siRNA group than in the PBS group (p<0.01), while the MMC group showed the highest blebs among three groups (p<0.01). The surgical eyes in both the siRNA and MMC groups had significantly larger bleb area compared with the PBS group (p<0.01), but there was no significant difference between the siRNA and MMC groups (p=0.214). There were no significant differences in IOP readings among the three groups on the designated days after surgery (all p>0.05). The histologic examination demonstrated that the eyes treated with siRNA showed a marked reduction in subconjunctival scar tissue compared with the eyes in the PBS group. The conjunctival epithelium appeared healthy without the acellularity that was present in the MMC group.

**Conclusions:**

Subconjunctival injection of cationic nano-copolymers mediated IKKβ targeting siRNA is associated with improved surgical outcome in a monkey model of trabeculectomy. This novel approach may potentially be a more controlled alternative as an anti-scarring agent in glaucoma filtration surgery.

## Introduction

The most common reason for glaucoma filtration surgery failures is the excessive subconjunctival scarring at the bleb and sclerostomy sites [1]. The introduction of the antimetabolites (e.g., mitomycin C [MMC] and 5-fluorouracil [5-FU]), has considerably improved the outcome of glaucoma filtration surgery in high-risk patients. However, they are relatively nonspecific and may be associated with an increased incidence of sight-threatening complications, such as either bleb leaks or endophthalmitis [2-4]. The challenge continues to be to develop a safe and effective method for modulating the wound healing process after glaucoma filtration surgery.

Nuclear factor kappa B (NF-κB) is an evolutionarily conserved family of DNA binding proteins that play a critical role in the regulation of genes involved in a variety of cellular processes [5]. They exist in cytoplasmic complexes with inhibitory proteins of the IκB family, and translocate to the nucleus to act as transcription factors when activated. IKKβ/IKBKB (IκB kinase beta), named after its function of phosphorylating IκB molecules, is the crucial kinase of the IKK-signalosome for activation of NF-κB by inflammatory stimuli, which is considered the classical (or canonical) pathway of NF-κB activation [6,7]. NF-κB has proven to be a ubiquitous factor associated with wound healing through the factor’s ability to stimulate transcription of various genes involved in the activation of inflammation and cell proliferation [8,9]. Given the central role of IKKβ in the NF-κB pathway, it may be assumed that pharmacological suppression of IKKβ, and subsequent blocking NF-κB activity, would be beneficial during the modulation of wound healing following glaucoma filtration surgery.

RNA interference (RNAi) was originally recognized as an evolutionary conserved defense mechanism in higher eukaryotic cells, and this system can easily and effectively inhibit the expression of one specific gene [10]. Recently, RNAi-mediated gene silencing has also demonstrated efficiency in mammalian cells, and this has led to the increasing feasibility of RNAi technology for the therapy of certain human diseases [11]. In a previous in vitro study [12], we determined the effects of IKKβ inhibition using RNAi technology on human Tenon’s fibroblasts (HTFs), which have a central role in the scarring process and filtration bleb failure. The small interfering RNA (siRNA) targeting IKKβ (IKKβ-siRNA) was designed and delivered into HTFs using a newly synthesized, ternary cationic copolymer called CS-*g*-(PEI-*b*-mPEG) as the vehicle. The results showed that the expression of IKKβ was downregulated, and the activation of NF-κB in the HTFs was subsequently inhibited. The proliferation of HTFs was effectively suppressed through the blocking of the NF-κB pathway. Furthermore, the non-viral siRNA carrier has indicated good biocompatibility, biodegradability, and transfection efficiency in vitro.

The purpose of this study was to investigate the effects of CS-*g*-(PEI-*b*-mPEG) mediated IKKβ targeting siRNA on the ocular wound healing process in a non-human primate model of filtration surgery, as well as to assess the safety and tolerance of this CS-*g*-(PEI-*b*-mPEG)/IKKβ-siRNA complex in vivo.

## Methods

### IKKβ-siRNA, CS-*g*-(PEI-*b*-mPEG), and CS-*g*-(PEI-*b*-mPEG)/IKKβ-siRNA complex

Three pairs of siRNA, which specifically targeted *IKKβ* (IKKβ-siRNA) were chemically synthesized by Ribobio Co. Ltd. (Guangzhou, China). siRNAs were derived from the coding sequence of the Rhesus Monkey (*Macaca mulatta*) *IKKβ* gene (GenBank XM_001096913), and were designed using an siRNA Target Finder program. A BLAST search checked all of the duplex sequences and target sequences of these siRNAs to preclude sequences with significant similarity to other genes in the *Macaca mulatta* genome. The duplex sequences of IKKβ-siRNA1, IKKβ-siRNA2, and IKKβ-siRNA3 were: 5′-GAA CAG AGG UUA AUA CAC AdT dT-3′ and 3′-dTd TCU UGU CUC CAA UUA UGU GU-5′, 5′-GAG CUG UAC AGG AGA CUA AdT dT-3′ and 3′-dTd TCU CGA CAU GUC CUC UGA UU-5′, and 5′-CCA AGA AGA GUG AAG AAC UdT dT-3′ and 3′-dTd TGG UUC UUC UCA CUU CUU GA −5′, respectively. The non-viral siRNA carrier, CS-*g*-(PEI-*b*-mPEG), and CS-*g*-(PEI-*b*-mPEG)/IKKβ-siRNA complex, were synthesized as previously described [12].

In our primary experiments, we found that IKKβ-siRNA2 (5′-GAG CUG UAC AGG AGA CUA AdT dT-3′ and 3′-dTd TCU CGA CAU GUC CUC UGA UU-5′) was the most effective among the three siRNAs in downregulating the transcription of *IKKβ* mRNA (data not shown). Therefore, we used IKKβ-siRNA2 as IKKβ-siRNA in the subsequent RNAi procedures.

### In vitro transfection and assays

Tissue explants of the rhesus monkey Tenon’s capsule were obtained from two monkeys (with no topical eye treatment). Tenon’s fibroblasts were cultured by a previously reported method [12], as described below. Cells were maintained as a monolayer at 37 °C with 5% CO_2_, 95% humidified atmosphere in DMEM supplemented with 10% FBS, 2 mM of L-glutamine, 100 IU/ml of penicillin, 100 μg/ml of streptomycin, and 25 μg/ml of amphotericin B. Cells between passages 3 and 6 were used for the following experiments.

Tenon’s fibroblasts were plated in six well plates with a density of 6x10^5^ cells per well, and incubated for either 12 h or 24 h (reaching 60%–70% confluence). Subsequently, the culture media were replaced with serum- and antibiotic-free DMEM 2 h before transfection. CS-*g*-(PEI-*b*-mPEG)/IKKβ-siRNA complexes were prepared, at an N/P ratio of 10, 30 min before transfection (based on our previous results), and cells were incubated with serum- and antibiotic-free DMEM that contained complexes corresponding to the determined final concentrations of IKKβ-siRNA (5, 10, 25, 50, and 100 nM) for 6 h. Then, the cells were maintained in the serum-supplied DMEM for either another 24 h or 48 h before the following assays were performed. Real-time RT–PCR, western blot, and cell proliferation assay were performed using the previously reported method [12]. Non-transfected fibroblasts and cells transfected with 100 nM of scrambled siRNA were regarded as the controls.

### In vivo studies

#### Animals

A total of 14 rhesus monkeys that weighed between 2 to 3 kg, and ranged in age from 3 to 4 years, were used in this study. A standard ophthalmologic evaluation was performed to exclude eye diseases. Experiments were conducted in accordance with the ARVO Statement for the Use of Animals in Ophthalmic and Vision Research.

#### Filtration surgery

Surgery was performed under general anesthesia with intramuscular injection of ketamine and local anesthesia with 0.5% proparacaine hydrochloride drops. Conventional trabeculectomy was performed in the superior quadrant. Briefly, after a lid speculum was positioned, a superior rectus bridle suture was used to rotate the eye inferiorly. A half-thickness, rectangular, 3x4-mm scleral flap was prepared after a limbus-based conjunctival flap was fashioned. A 1x2-mm sclerostomy was followed by peripheral iridectomy. The scleral flap was closed with two 10–0 nylon sutures at each corner. The conjunctiva was closed with a continuous locking suture. Antibiotics and steroids were applied topically after surgery. The surgery was executed by the same masked individual.

#### Treatment regimen

The animals were randomly allocated to one of three treatment regimens: subconjunctival injection of either CS-*g*-(PEI-*b*-mPEG)/IKKβ-siRNA (six eyes) or phosphate buffered saline (PBS; four eyes), or treated with MMC (four eyes). In the IKKβ-siRNA group, 0.1ml of CS-*g*-(PEI-*b*-mPEG)/IKKβ-siRNA (dissolved in RNase-free H_2_O, 50 nM concentration) was injected subconjunctivally into the bleb area by a 30-gauge needle, after the conjunctiva was closed at the time of surgery. The needle was placed 5 mm behind the limbus, at the nasal margin of the superior rectus muscle. The subconjunctival injection was repeated on day 7 after surgery, under general and topical anesthesia. A similar volume of PBS was administered in the same manner as the negative control. The MMC group was used as the positive control. Eyes were treated at the time of surgery with a solution of 0.2 mg/ml MMC, using a section of soaked microsponge, which was placed between the conjunctiva and sclera over the determined filtration site for 5 min. The area was then thoroughly irrigated with physiologic saline.

#### Clinical observations

The slit-lamp observations were performed twice per week for the first four weeks, and once per week thereafter, until the animals were executed on day 60 to assess the conjunctival hyperemia, corneal edema, either intraocular inflammation or hemorrhage, and anterior chamber depth. The eyes were also stained with fluorescein to look for evidence of either corneal epithelial toxicity or bleb leak. Corneal endothelial cell density was examined preoperatively and 1 month after surgery. Bleb characteristics, including length and width, were measured with calipers, and height was graded semiquantitatively by slit-lamp examinations (0, flat; 1, shallow/formed <1 mm; 2, elevated <2 mm; 3, high >2 mm). Bleb failure was defined as the appearance of a flat, vascularized, scarred bleb in association with a deep anterior chamber. Intraocular pressure (IOP) was measured (average of three consecutive readings) at the same time intervals using an applanation tonometer (Tonopen; Reichert, Inc., Depew, NY). All experimental procedures and analyses were conducted by the same masked investigator.

#### Histologic analysis

Two monkeys in each group were killed on day 60. The eyes were enucleated and prepared for histological examination by conventional optical microscopy. Serial sections were cut through the sclerostomy site, and stained with standard hematoxylin-eosin.

### Statistical analysis

The statistical analyses were conducted using SPSS version 13.0 for Windows (SPSS Inc., Chicago, IL). The in vitro results were analyzed by Student's *t*-test. Survival analysis was performed for bleb failure using the Kaplan–Meier log rank test. The area and height of the bleb were analyzed with a generalized linear model (GLM; SPSS 13.0) repeated-measures procedure to compare the two groups. IOP measurements were compared among groups using a one-way ANOVA. P-values of less than 0.05 were considered significant.

## Results

### In vitro studies

#### Downregulating effect on *IKKβ* expression

**Figure 1:**
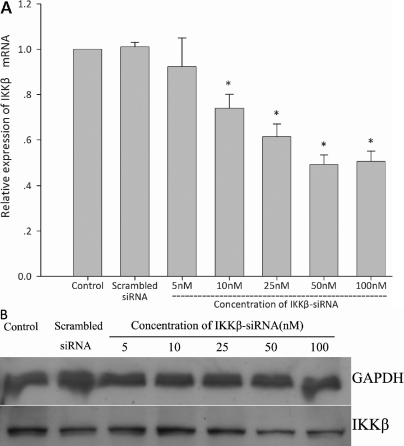
IKKβ-siRNA inhibits the expression of *IKKβ* at both the mRNA and protein levels in vitro. **A**: mRNA transcription of *IKKβ* in monkey Tenon’s fibroblasts assessed by real-time RT–PCR 24 h after 5–100 nM IKKβ-siRNA were transfected. The normalized *IKKβ* mRNA level of nontransfected fibroblasts (control) is taken as 1.0 (the asterisk indicates a p<0.05, mean±SD, n=3). **B**: Protein levels of IKKβ are demonstrated by western blot.

Real-time PCR assay revealed that mRNA transcription of *IKKβ* in monkey Tenon’s fibroblasts was suppressed in a dose-dependent manner 24 h after 5 to 100 nM of IKKβ-siRNA were transfected (Figure 1A). Significant inhibition (26%) was detected following transfection of 10 nM of IKKβ-siRNA compared to the control group (p<0.05), and maximum suppression (51%) was observed in the group transfected with 50 nM of IKKβ-siRNA. Meanwhile, the expression of IKKβ protein was also inhibited in a dose-dependent manner after IKKβ-siRNA transfection into the fibroblasts. Alternatively, no significant difference was found in the expression of *GAPDH* between the control group and groups transfected with IKKβ-siRNA (Figure 1B).

#### Inhibiting the proliferation of monkey Tenon’s fibroblasts

**Figure 2:**
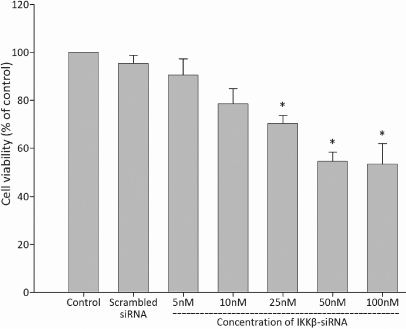
The inhibition effect of IKKβ-siRNA on the proliferation of monkey Tenon’s fibroblasts. Data are presented as the percentage of viable cells compared with the untreated (control) cells (mean±SD, n=6). An asterisk indicates that p<0.05.

The RNAi process that targets *IKKβ* repressed the proliferation of monkey Tenon’s fibroblasts in an siRNA dose-dependent manner in vitro. The cell viability of Tenon’s fibroblasts transfected with more than 25 nM of IKKβ-siRNA showed significant differences compared with those of the control group (p<0.05), as shown in Figure 2. However, the proliferation of Tenon’s fibroblasts transfected with 100 nM of scrambled siRNA was not affected.

### In vivo studies

Surgical eyes exhibited slight hyperemia of the conjunctiva, reversible edema of the cornea, and measurable flare and cells in the anterior chamber, which all resolved within the first week post-surgery. The mean endothelial cell density measured 1 month after surgery decreased slightly compared with preoperative values, but this change was not statistically significant in all groups (Table 1). In the experimental group, repeated subconjunctival injections of CS-*g*-(PEI-*b*-mPEG)/IKKβ-siRNA were well tolerated in the monkeys. There were no remarkable differences in ocular findings between the control and experimental groups. Superficial punctuate keratitis occurred in 1 eye of the MMC treated group. Not one case of bleb leaks or endophthalmitis was observed.

**Table 1:** The mean endothelial cell density (mean±SD) before and after surgery.

**Group**	**siRNA group**	**PBS group**	**MMC group**
Presurgery	3,498±338	3,434±642	3,453±418
Postsurgery	3,388±150	3,362±499	3,209±146
	p=0.343	p=0.594	p=0.291

#### Bleb survival and characteristics

**Figure 3:**
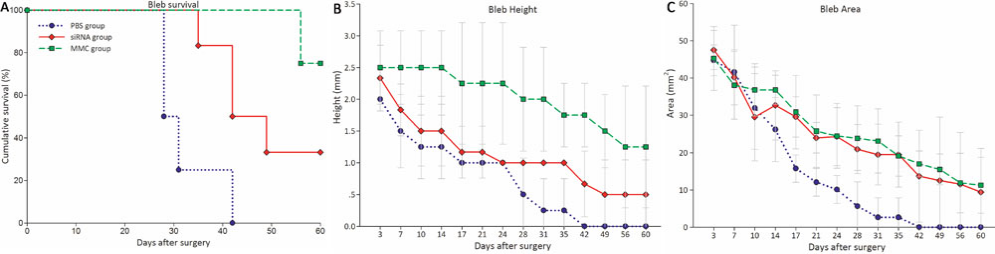
The effects of IKKβ-siRNA, MMC, and PBS on bleb survival, bleb height, and bleb area. **A**: IKKβ-siRNA (n=6) and MMC (n=4) groups significantly prolonged bleb survival compared with the PBS group (n=4) in the monkey model (log rank test; p<0.01), as shown in the Kaplan-Meier survival curve. **B**: Higher blebs were observed in the siRNA group than in the PBS group (p<0.01), while the MMC group showed the highest blebs among the three groups (p<0.01). **C**: Eyes in the siRNA and MMC groups had significantly larger bleb area compared with the PBS group (p<0.01), but there was no significant difference between the siRNA and MMC groups (p=0.214).

IKKβ-siRNA and MMC groups significantly prolonged bleb survival compared with the PBS group in the monkey model (log rank test; X^2^=11.548, p<0.01), as shown in the Kaplan–Meier survival curve in Figure 3A. The medians for survival days were: 45.5, 60, and 29.5 in the siRNA, MMC, and PBS groups, respectively.

**Figure 4:**
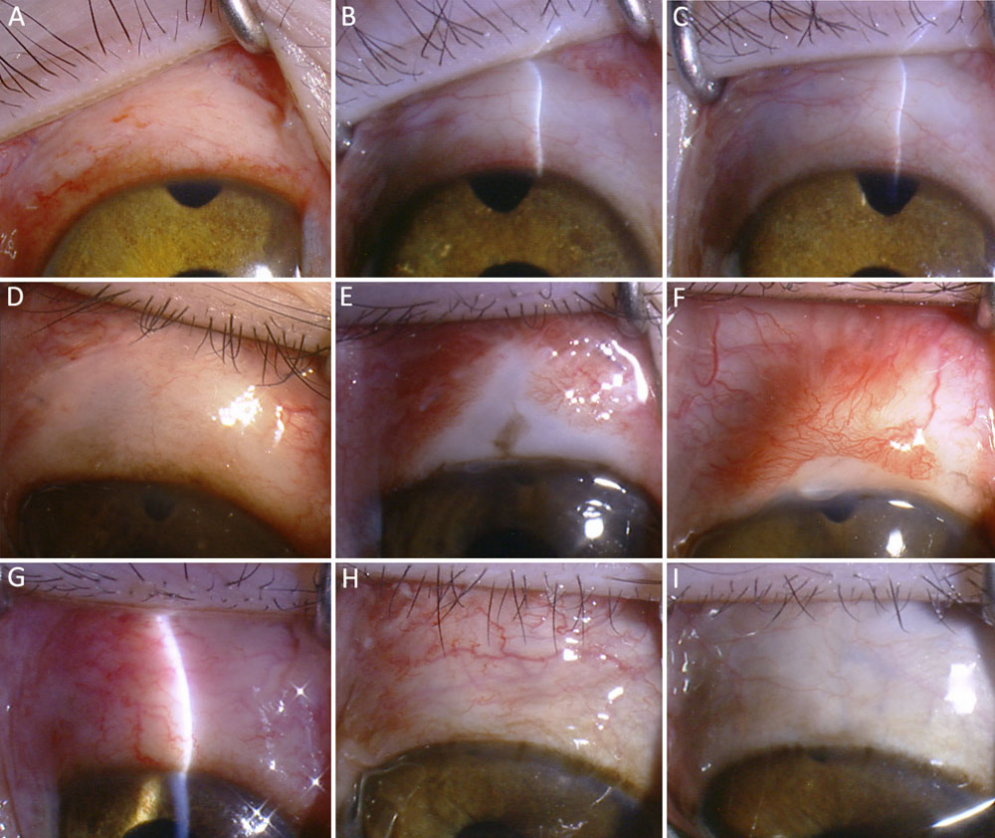
Figure 4. Representative bleb photographs after surgery. **A**-**C**: siRNA group, **D**-**F**: MMC group, **G**-**I**: PBS group, on day 3, day 21, and day 49, respectively. Treatment with IKKβ-siRNA was associated with an elevated, diffuse, fleshy-looking bleb compared with the flat, scarred bleb in the PBS group (day21 and day 49). MMC treated eyes were associated with typical avascular cystic blebs surrounded by local conjunctival hyperemia while IKKβ-siRNA resulted in a relatively less-elevated bleb and a normal-appearing conjunctiva.

Height and area of the blebs in the surgical eyes were compared among the groups. Eyes in the siRNA and MMC groups had significantly higher and larger blebs than the PBS group (F=97.947 and 34.364, respectively, both p<0.01; Figure 3B,C). The blebs in the MMC group were higher than blebs in the siRNA group (p<0.01), while there was no significant difference in the area of the blebs between two groups (p=0.214). Figure 4 depicted the typical appearance of the blebs post-surgery in the three groups. MMC treated eyes were associated with typical avascular blebs surrounded by a local conjunctival hyperemia. Subconjunctival injection of IKKβ-siRNA, however, resulted in a relatively less elevated bleb and a normal-appearing conjunctiva.

#### Intraocular pressure

**Figure 5:**
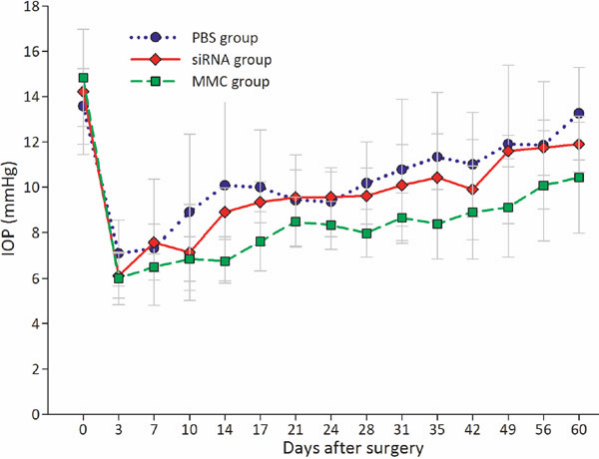
The effects of IKKβ-siRNA, MMC, and PBS on postoperative IOPs. There were no significant differences in IOP readings among the three groups on the designated days after surgery (n=6, n=4, and n=4, respectively, all p>0.05).

Table 2 and Figure 5 both illustrated the IOP change after filtration surgery among the three groups. Before the surgery, there was no significant difference in the baseline IOP (F=0.284, p=0.758). A steep reduction in IOP was observed in the early stages after surgery. Thereafter, IOP gradually increased in all groups. Although there was a trend toward decreases in IOPs for eyes treated with siRNA and MMC on the designated days after surgery, these data were variable and not significantly different from those of eyes treated with PBS (all p>0.05).

**Table 2:** Summary table of the mean IOPs (mmHg±SD) in each group before and after surgery.

**Days after surgery**	**PBS group**	**siRNA group**	**MMC group**
0	13.58±1.67	14.22±2.77	14.83±2.14
3	7.10±1.45	6.12±1.00	6.00±1.15
7	7.33±1.04	7.58±2.78	6.50±0.58
10	8.90±3.44	7.13±2.11	6.85±0.98
14	10.08±4.19	8.90±1.07	6.75±0.96
17	10.00±2.53	9.33±0.90	7.63±1.30
21	9.43±2.00	9.55±1.22	8.48±1.09
24	9.35±1.51	9.57±1.11	8.33±1.04
28	10.18±1.82	9.62±1.23	7.98±1.04
31	10.78±3.10	10.08±1.80	8.65±1.11
35	11.33±2.85	10.43±1.93	8.38±1.53
42	11.00±2.31	9.90±2.21	8.90±2.04
49	11.90±3.50	11.60±0.69	9.10±2.16
56	11.85±2.81	11.75±1.22	10.08±2.43
60	13.25±2.04	11.90±1.24	10.43±2.45

#### Histologic examination

**Figure 6:**
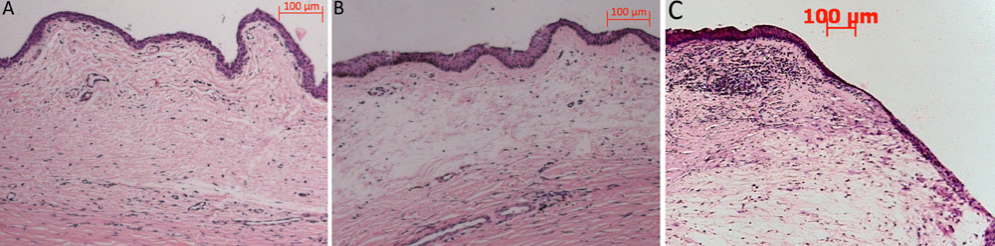
Histologic characteristics of the surgical site associated with the PBS group, siRNA group, and MMC group on day 60 after surgery. Treatment with IKKβ-siRNA (**B**) showed a marked reduction in subconjunctival scar tissue compared with the PBS group (**A**). The conjunctival epithelium appeared healthy, and the subepithelial connective tissue was loosely arranged, with moderate subconjunctival cavities, while the MMC treated eyes (**C**) exhibited marked acellularity and large bleb cavities.

The histological sections of the surgical sites were examined at the end of the postoperative period. Eyes treated with PBS exhibited massive fibrosis, and were accompanied by nearly complete scarring over the sclerostomy site (Figure 6A). In contrast, eyes treated with siRNA showed a marked reduction in subconjunctival scar tissue (Figure 6B). The conjunctival epithelium appeared healthy, and the subepithelial connective tissue was loosely arranged and had moderate subconjunctival cavities. As expected, eyes treated with MMC exhibited marked acellularity and large bleb cavities (Figure 6C). The thinning of the conjunctiva and Tenon’s layer were present in the region of the surgery.

## Discussion

Glaucoma filtration surgery often fails due to excessive scarring, which occurs during the wound healing process. The perioperative administration of antimetabolites has significantly improved the success rate of filtration surgery. However, they are associated with an increased incidence of sight-threatening complications, such as bleb leaks and endophthalmitis [2-4]. These problems have stimulated the search for new agents to minimize ocular complications.

Since the discovery of NF-κB in 1986, inappropriate activation of NF-κB is thought to be linked to a great variety of diseases and pathological states, including tissue injury, inflammation, and repair [5,8]. As IKK integrates many NF-κB-activating pathways, the most effective and specific approach to the modulation of NF-κB activity may result from IKK inhibitors [9]. In a previous study [12], the inhibiting effects of NF-κB blocking by IKKβ-siRNA on the cultured human Tenon’s fibroblasts were determined. We extend the investigation of IKKβ-siRNA in a non-human primate model of glaucoma surgery in the current study. Because of the highly specific nature of RNA interference, we first re-designed and screened the highly effective IKKβ-siRNA, based on the coding sequence of the *Macaca mulatta IKKβ* gene. The in vitro results showed that the inhibiting effects were dose-dependent and reached a plateau at the concentration of 50 nM. Previous studies have reported that the duration of siRNA induced silencing may last approximately 5–7 days, and wound healing at the sclerectomy site by proliferating fibroblasts occurs within the first 14 postoperative days [13,14]. Therefore, we used subconjunctival injection of 50 nM concentration of IKKβ-siRNA into the bleb area at the time of surgery, and repeated injection on day 7 post surgery in the present study.

The results indicated that the IKKβ-siRNA treated eyes exhibited prolonged bleb survival and delayed increase of IOP postoperatively compared with PBS control eyes. Histologic analysis of the surgical sites also showed that both IKKβ-siRNA and MMC prevented fibrosis, but IKKβ-siRNA treated eyes appeared less destructive to local tissue. The mechanism of IKKβ-siRNA preventing fibrosis may be related to the inhibitory effect of NF-κB blocking on inflammation and the proliferation of Tenon’s fibroblasts. Other studies have also reported that the inhibition of NF-kB using *IKKβ* inhibitors ameliorated the pathogenesis in many fibrotic diseases, including lung fibrosis [15-17], hepatic fibrosis [18,19], and skin proliferative disorders [20,21]. On the other hand, NF-κB signaling occurs through either the classical (canonical) or the alternative (non-canonical) pathway. Whereas classical NF-κB activation is IKKβ dependent and closely linked to the orchestration of inflammatory responses, the alternative pathway depends on IKKα homodimers and NF-κB inducing kinase (NIK), and it is particularly important for B-cell maturation and immune responses [22]. Since NF-κB is also involved in normal cellular physiology, global inhibition of NF-κB may result in profound side effects, such as the apoptosis of healthy cells. However, selective suppression of inflammation-induced NF-κB activity by IKΚβ-siRNA may leave some NF-κB-dependent signaling and is beneficial for wound modulation [9,22]. We propose this is the reason for the more effective tissue preservation of IKKβ-siRNA than that of MMC. This also may be one of the reasons that the inhibitory effect of IKKβ-siRNA did not increase when the concentration of IKKβ-siRNA was increased more than 50 nM in vitro in our study (another reason may be due to the limitation of the efficiency of transfection). This novel approach, based on both the IKKβ-dependent NF-κB pathway and RNAi technology, may potentially be a more controlled alternative as an anti-scarring agent in glaucoma filtration surgery.

The eye is a relatively isolated compartment, which makes it an ideal target organ for gene therapy [23]. The main advantage of RNAi is its ability to silence the expression of deleterious genes of known sequence. There has been rapid progress toward its use as a therapeutic modality since its first description less than a decade ago. Nakamura et al. [24] and Wang et al. [25] have already reported improved outcomes of glaucoma surgery in animal models using RNAi technologies. Both of the studies used viral vectors to deliver the gene of interest. Although having the advantage of high transfection efficacy, the use of viral delivery systems is limited by endogenous recombination and host immunity [26]. Moreover, since ontogenesis and mortality have been reported [27], concerns have been raised regarding the safety of using viral vectors in gene therapy trials in humans. In this study, we used a novel biodegradable copolymer, CS-*g*-(PEI-*b-*mPEG) as the vector. The in vitro results have indicated that CS-*g*-(PEI-*b-*mPEG) featured good biocompatibility and transfection efficiency. Subconjunctival IKKβ-siRNA/CS-*g*-(PEI-*b-*mPEG) application in the animal model appeared clinically safe and well tolerated within the experimental time. Furthermore, it has been proposed that the hydrophobic domains in the CS-*g*-(PEI-*b-*mPEG) may be capable of storing hydrophobic drugs and allowing a slow and sustained release [28], which may reduce both the need for frequent applications and toxic ocular side effects.

In this study no significant differences were detected in IOP among the three groups. There are questions about the usefulness of IOP as a measure of successful filtration surgery and bleb function in the animal model. Several investigations with the normotensive model failed to find the IOP changes after antiproliferative treatments, in spite of effects detected on the bleb morphologic characteristics [29-31]. Therefore, bleb survival rather than IOP was used as the primary outcome measure in the current study [30,31].

In summary, the current study demonstrated that inhibition of NF-κB activity by local application of IKKβ-siRNA improved the surgical outcome in a non-human primate model of glaucoma filtration surgery. Further studies are needed to improve the application methods, and to investigate the long-term effects and safety of this novel anti-scarring agent.
